# Hypergravity and ERK Inhibition Combined Synergistically Reduce Pathological Tau Phosphorylation in a Neurodegenerative Cell Model

**DOI:** 10.3390/cells14141058

**Published:** 2025-07-10

**Authors:** Valerio Mignucci, Ivana Barravecchia, Davide De Luca, Giacomo Siano, Cristina Di Primio, Jack J. W. A. van Loon, Debora Angeloni

**Affiliations:** 1 Graduate School in Translational Medicine, Scuola Superiore Sant’Anna, Via G. Moruzzi, 1, 56124 Pisa, Italy; valerio.mignucci@unitn.it (V.M.); davidedeluca@cnr.it (D.D.L.); 2 The Institute of Biorobotics, Scuola Superiore Sant’Anna, Via G. Moruzzi, 1, 56124 Pisa, Italy; i.barravecchia@santannapisa.it; 3 Institute of Neuroscience, National Research Council of Italy, Via G. Moruzzi, 1, 56124 Pisa, Italy; giacomo.siano@in.cnr.it (G.S.); cristina.diprimio@in.cnr.it (C.D.P.); 4 Department of Oral and Maxillofacial Surgery/Oral Pathology, Amsterdam UMC Location VUmc and Academic Center for Dentistry Amsterdam (ACTA), Vrije Universiteit Amsterdam, 1081 HV Amsterdam, The Netherlands; jjwavanloon@gmail.com; 5 European Space Research and Technology Centre–Directorate of Technology, Engineering and Quality–Materials and Processes Section Laboratory (ESTEC, TEC-MMG-Lab), European Space Agency (ESA), 2201 AZ Noordwijk, The Netherlands; 6 Health Science Interdisciplinary Center, Scuola Superiore Sant’Anna, Via G. Moruzzi, 1, 56124 Pisa, Italy

**Keywords:** European Space Agency (ESA), hypergravity, large diameter centrifuge (LDC), neurodegeneration, PD-0325901, tauopathies

## Abstract

This study evaluates the effects of hypergravity (HG) on a neurodegenerative model in vitro, looking at how HG influences Tau protein aggregation in Mouse Hippocampal Neuronal Cells (HT22) induced by neurofibrillary tangle seeds. Overall, 50× *g* significantly, synergistically, reduced the Tau aggregate Area when combined with ERK-inhibitor PD-0325901, correlating with decreased phosphorylation at critical residues pS262 and pS396. These findings suggest HG treatments may help mitigate cytoskeletal damage linked to Tau aggregation.

## 1. Introduction

The space age has unveiled significant insights into how gravity impacts human physiology, particularly for astronauts on long-duration missions who encounter environmental stressors (i.e., acceleration, vibrations, microgravity, and radiation) that can negatively affect both the structure and function of the brain, potentially leading to cognitive deficits [1] and long-lasting changes [2,3,4]. Research using mice indicates that cosmic radiation may promote amyloid-β accumulation, neuroinflammation, and cognitive dysfunction related to the hippocampus [5,6]. Simulated microgravity (MG) disrupts protein clearance, with various ground simulation studies on rhesus macaques and rats revealing pathological changes ([7] and references therein). Additionally, gravity influences protein aggregation, suggesting a direct role of physical forces in this process [8,9]. Furthermore, the literature suggests that spaceflight may accelerate brain aging, like its established effects on cardiovascular and musculoskeletal systems [6,10]. These findings raise crucial concerns for astronaut health and also offer insights into neurodegenerative mechanisms that could be relevant for both space and terrestrial biomedicine [11,12].

Neurodegenerative diseases, which are more common with age and for which there are no cures [13], encompass a range of disorders, including tauopathies characterized by the accumulation of Tau protein in neurons [13,14,15]. The hyperphosphorylation of Tau leads to its detachment from microtubules, forming neurofibrillary tangles (NFTs) that disrupt cellular functions and cause neuronal death [13,16]. Synthetic forms of NFTs, called Tau seeds (TSs), behave as seeds in promoting neurodegeneration when introduced to cells in vitro, with phosphorylation by kinases like Extracellular signal-Regulated Kinase (ERK) being critical for aggregate formation [13]. Kinase inhibitors, including those approved for cancer treatment, may also be effective for Tau-related pathologies. One such inhibitor, PD-0325901 (PD-901), has demonstrated promise in reducing Tau aggregate formation in a neurodegenerative model [17].

Changes in gravitational force affect the cytoskeleton across different cell types ([18,19], and therein). For example, human neuroblastoma cells SH-SY5Y exhibit distinct behaviors when exposed to MG versus hypergravity (HG).

This work, although preliminary, explores the possibility of applying centrifuge therapy to the treatment of neurodegeneration at large. Centrifuge therapy has already been in clinical practice for several decades now for a number of human conditions, including peripheral artery disease, coronary artery disease, lymphedema, complex regional pain syndrome, secondary Raynaud’s phenomenon, and systemic sclerosis [20]. In human research and application, one of the very early predecessors of the current short arm centrifuges was probably Cox’s chair, described more than two centuries ago in his book “Practical Observations on Insanity” [21]. Also, the later work by Halloran and his application of a circulating swing used in clinical medicine in the treatment of mental health issues was the basis for rotating devices in human medicine [22]. More recently, a case study reported improvement in mobility and changes in the electro-encephalogram (EEG) of a patient with secondary progressive multiple sclerosis following multiple, mild HG sessions [23]. Observations from space medicine suggest that artificial gravity training is a promising avenue for physical rehabilitation, especially in counteracting musculoskeletal decline due to microgravity and inactivity. HG has also been explicitly proposed as a potential therapeutic approach specifically against neurodegeneration [24], although the molecular mechanisms underlying these effects remain largely unexplored.

In our previous gravitational biology studies [18,19], we could observe the cytoskeletal adaptation changes induced by HG in a cell model in vitro. Since neurodegenerative pathologies do involve cytoskeletal damage [25,26], based on the above, we decided to test the hypothesis whether or not HG had an effect on a neural cell model of neurodegeneration associated with Tau protein deposition and neurofibrillary tangles formation in vitro.

Therefore, in this study, we investigated the effects of HG, also combined with the ERK inhibitor PD-901, on Tau aggregates in the hippocampal neuronal cell line HT22. Our findings indicated that HG does not compromise cell viability; the combination of HG and PD-901 led to a synergistic reduction in the size of Tau aggregates, correlating with decreased phosphorylation at pS262 and pS396, both associated with neurodegeneration progression, while leaving pT231 unaltered. Utilizing the Large Diameter Centrifuge (LDC) of the European Space Agency (ESA, Noordwijk, The Netherlands, Supplementary Scheme S1), we observed a threshold effect from HG treatments.

Understanding how changes in gravitational forces affect neuronal behavior could provide valuable insights into the mechanisms of the disease and possibly new therapeutic tools.

## 2. Materials and Methods

### 2.1. Cell Culture

HT-22 cells (Mouse Hippocampal Neuronal Cell Line, Sigma-Aldrich, St. Louis, MO, USA) were cultured in Dulbecco’s Modified Eagle’s Medium (DMEM) Low-Glucose (Sigma, St. Louis, MO, USA) complemented with 10% Fetal Bovine Serum (FBS, Gibco, Grand Island, NY, USA), 200 mM L-Glutamine (Sigma-Aldrich), and 1% Penicillin/Streptomycin (Sigma-Aldrich) in ventilated plastic flasks (Sarstedt, Nümbrecht, Germany) at 37 °C, 5% CO_2_. Cells were split every 48 h, at about 80% confluency, using 1X Trypsin/EDTA (Sigma-Aldrich) in 1X Phosphate Buffer Saline (PBS).

Live cells were shipped from Italy to the LDC facility (Supplementary Scheme S1) in 1M HEPES, pH 7.4 (Sigma-Aldrich), and added to the complete medium. For HG treatments, 20,000 cells were seeded on day 1 in Nunc Lab-Tek Flask on Slide, and from there on flaskettes with glass bottom, for microscopy analysis (ThermoFisher Scientific, Waltham, MA, USA). To improve cell adhesion, flaskettes were treated with Poly-D-Lysine (Sigma-Aldrich) for one hour at 37 °C, washed with sterile water, and let dry under the laminar-flow hood for at least three hours, at RT. During HG protocols, cells were kept at 37 °C within an incubator placed inside a gondola of the LDC.

Following data acquisition, cells were fixed with ice-cold methanol at RT for 3 min and then stored in 1X PBS at 4 °C.

### 2.2. Transfection Protocols

The plasmid encoding the CST^P301S^ was transfected in an antibiotic-free medium, 24 h after cell seeding. Recombinant heparin-assembled P301S Tau fibrils were prepared as in [17]. Tau fibrils were delivered to cells at a concentration of 0.3 µg/cm^2^. Transfection protocols were performed with Lipofectamine 2000 (ThermoFisher, Waltham, MA, USA) diluted in DMEM-LG without supplements. Two hours after transfection, DMEM-LG complete was added without removing the transfection solution.

### 2.3. Drug Treatment

Cells were exposed to 1 µM PD-0325901 (PD-901, for brevity) in DMSO (Sigma-Aldrich) or DMSO as the vehicle. Pharmacological treatment was performed 72 h after cell seeding and lasted 72 h without any medium refresh.

### 2.4. HG Protocols

HG is a circumstance in which samples experience an acceleration higher than gravitational acceleration on the Earth’s surface (1× *g* = 9.8 m/s^2^). Centrifuges produce HG environments, resulting from the sum of centrifugal acceleration and gravitational acceleration vectors. Two types of centrifuges were used for this study: the Large Diameter Centrifuge (LDC) of the European Space Research and Technology Center (ESTEC) of ESA (Noordwijk, The Netherlands), during the “Spin your Thesis!” (SYT!) 2020 campaign (Supplementary Scheme S1), where cells were stimulated at 10× *g* and 20× *g*, for 3 h. In the home laboratory, we used the 5804 bench-top centrifuge (Eppendorf, Hamburg, Germany), a classic bench-top centrifuge that holds the swing-out rotor (A-2-DWP) and allows the insertion of cell plates. In the home lab, cells were exposed to a nominal value of 50× *g*. Overall, 50× *g* was chosen as the lowest setting of the home centrifuge that allows us to obtain consistent biological results.

We took into account the possible effect of inertial shear forces in the home lab standard centrifuge. To minimize the effect, we used a swing-out rotor, which should ensure that F*g* is administered perpendicularly to the surface of the cell culture at regime during the run [27]. Also, we used relatively small wells (3 cm diameter) to minimize the inertial shear force that increases laterally from the center of centrifugation. To minimize unwanted forces and ensure maximal consistency, we performed all experiments using a single glass-bottom plate (3 cm diameter) fixed to the center of the rotor rack (Supplementary Scheme S2).

### 2.5. Viability Assays

To evaluate the effects of chemical compounds and/or HG, cell viability was evaluated either with manual counting (using Trypan blue dye exclusion) or with the MTT Assay (Sigma-Aldrich), according to manufacturer instructions. Overall, 20,000 cells were seeded per one chamber slide (ThermoFisher); the next day, cells were either placed in the centrifuge (50× *g*, RT, 3 h), or left in the incubator (1× *g*, 37 °C, 3 h), or placed over the centrifuge lid (1× *g*, RT, 3 h), as reference samples. Cells were counted 24 and 48 h after HG.

### 2.6. Immunofluorescence (IF) Assay and Fluorescence Staining

Cells were cultured in chamber slides (ThermoFisher) and in WillCo dishes (Willcowells, Amsterdam The Netherlands). Cells were fixed with either 4% paraformaldehyde (PFA) or ice-cold methanol. After three washes with 1X phosphate buffer solution (PBS), cells were permeabilized with PBS-Triton 0.1% for 10 min at RT, then blocked with 2% bovine serum albumin (BSA) for 60 min. Primary antibodies were added for 60 min at 37 °C, in humid chamber (α-tubulin, 1:500, ThermoFisher). After three washes with 1X PBS-Tween 0.1%, secondary antibody (Anti-mouse, 1:500, Life Technologies, Waltham, MA, USA), or Phalloidin Atto 550 (1:350, Sigma-Aldrich), and DAPI (1:1000, Sigma-Aldrich) were added for 60 min at RT. After three washes with 1X PBS-Tween 0.1%, samples were finally mounted with glass coverslips in Aqua PolyMount (Polysciences, Warrington, PA, USA).

### 2.7. Western Blot

Total cell extracts were prepared in lysis buffer supplemented with protease and phosphatase inhibitors. For each sample, we loaded 20 μg of each fraction. Proteins were separated by 8% acrylamide gel and electroblotted onto nitrocellulose membranes, Hybond-C-Extra (Amersham Biosciences, Piscataway, NJ, USA). Membranes were blocked for 5 min (EveryBlot Blocking Buffer, Biorad, Hercules, CA, USA) and incubated with the primary antibody (overnight, 4 °C) and with horseradish peroxidase (HRP)-conjugated secondary antibodies (1 h, RT). Membranes were incubated for 1 min with Amersham ECL Detection Reagent (Cytiva, Marlborough, MA, USA) or SuperSignal West (ThermoFisher Scientific), and immunoblot images were acquired by ChemiDoc Imaging System (Biorad) (acquisition setup: Auto and Quality exposure). Primary antibodies were as follows: mouse anti-Tau (Tau13) 1:1000 (SantaCruz, Dallas, TX, USA); rabbit anti-pTau S262 1:500 (Thermo Fisher Scientific); rabbit anti-pTau S396 1:500 (Thermo Fisher Scientific); rabbit anti-pTau T231 1:500 (Thermo Fisher Scientific); mouse anti-GAPDH 1:15,000 (Fitzgerald, North Acton, MA, USA).

### 2.8. Data Acquisition and Analysis

Cell imaging was performed under the Nikon Ti2 microscope (Nikon, Melville, NY, USA), with objective Nikon Plan APO lambda 60X/1.40 Oil. IF assays on fixed cells were analyzed under Nikon Ti2 microscope with the objective Nikon Plan APO lambda 60X/1.40 Oil, equipped with Mono Camera Nikon DS-Fi3.

Images were analyzed with ImageJ 1.53 e. The dimensions and shape of cells or nuclei were obtained by drawing the contour of cells or nuclei. The Area of cells and nuclei was obtained by calculating how many pixels were present within the contour line and then converted to μm^2^. Circularity was calculated according to the formula 4π × [Area]/[Perimeter]^2^. Roundness was calculated according to the formula 4 × [Area]/(π × [Major axis]^2^).

The Plot Profile algorithm (ImageJ suite) was used to analyze the cytoskeleton. A segment of fixed dimension was drawn across a single cell or nucleus, and we calculated the intensity of fluorescence along every single pixel of the segment.

### 2.9. Statistics

Statistical analyses were performed with GraphPad software v10 (Prism). Specific tests are indicated in the figure legends.

## 3. Results

**Figure 1:**
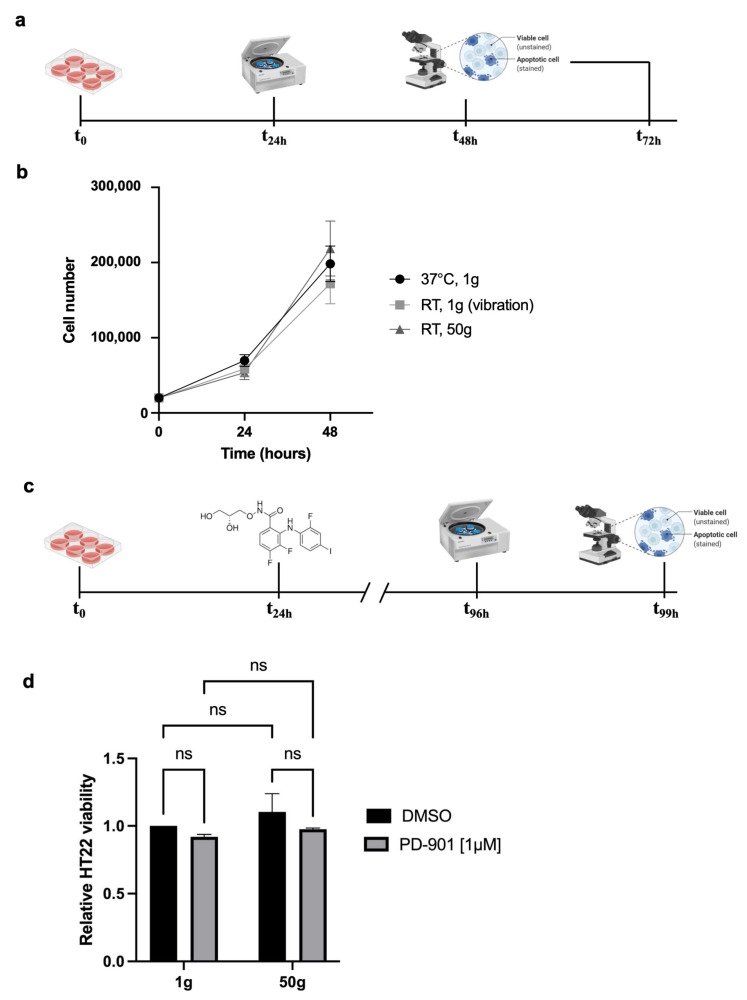
Mouse Hippocampal Neuronal Cells HT22 endure exposure to HG and ERK inhibitor PD-901 both alone and combined. (**a**) 20,000 cells per group were seeded at T_0_; the next day, cells were exposed to a gravitational acceleration of 50× *g* for three hours, at RT. Imaging and viability assays were performed 24 and 48 h later. (**b**) Viability assays of the three groups of samples: one centrifuged at 50× *g* (triangles), and two reference groups: one left in the cell incubator (circles), one kept at RT on the centrifuge lid (squares) to sense the vibrations of the operating instrument. Data represent mean ± SEM. Statistical test: Two-way ANOVA, *n* = 3. (**c**) Experimental design as in A with the adjunctive step of drug administration, 1μM PD-0325901 (PD-901). (**d**) Viability assay of HT22 cells exposed to 50× *g* with 1μM PD-901 (gray) or without (Dimethyl sulfoxide, DMSO, as the vehicle, black). Data represent mean ± SEM. Statistical test: Two-way ANOVA, *n* = 3, ns = not significant, *p* > 0.05. Note: ‘*n* = 3’ is an indication of the number of biological replicas (i.e. number of experiments), each one having its own three technical replicas.

We studied the effects of HG on a neuronal cell model of tauopathy, beginning by testing the feasibility of exposing HT22 cells to 50× *g* (Figure 1a). Throughout the study, we employed two control groups: one stayed under standard culture conditions in the cell incubator at 37 °C, the other was placed on top of the centrifuge lid at room temperature (RT) during the centrifugation, to be subjected to the centrifuge vibrations without exposure to HG.

The results showed no significant differences in cell viability among the conditions tested (Figure 1b), suggesting the HT22 cells also tolerated 50× *g* when combined with the ERK inhibitor PD-901 (experimental design in Figure 1c). The feasibility of double stimulation (Figure 1d) allowed us to proceed with further experiments, based on the observation that HG (50× *g*) alone or combined with PD-901 does not affect HT22 viability.

**Figure 2:**
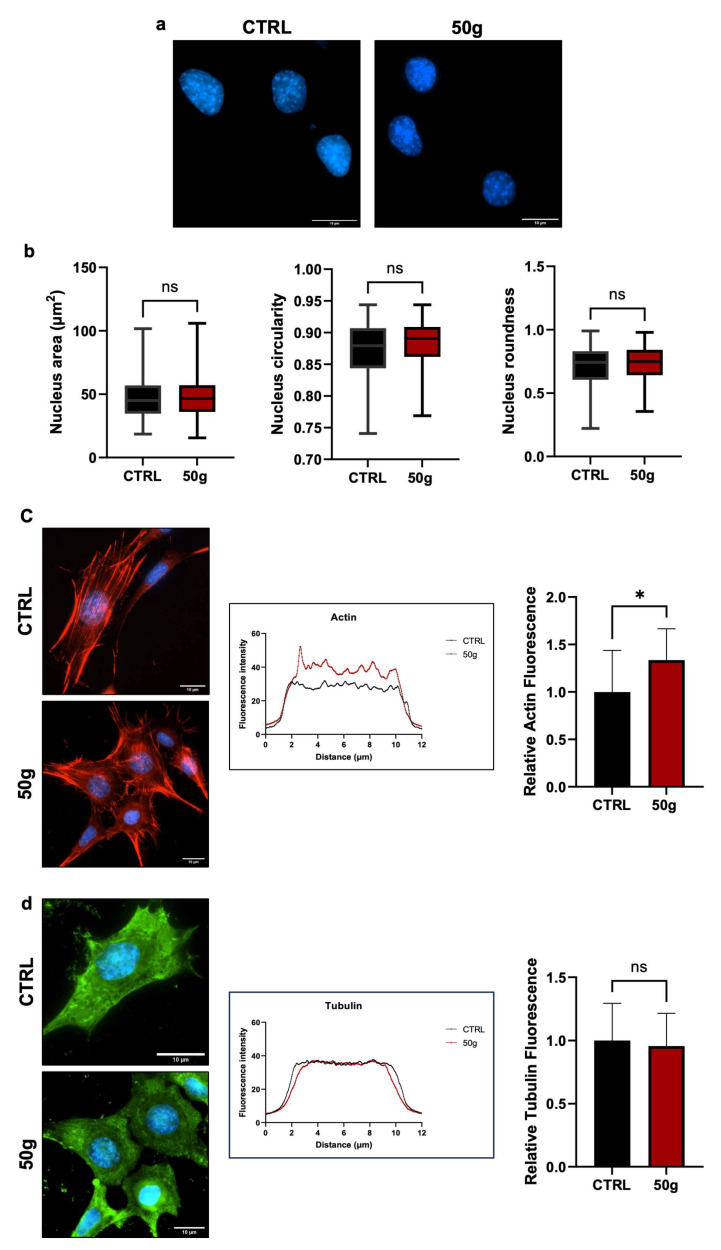
HG does not change Area, Circularity, and Roundness of HT22 nuclei; however, it modifies the organization of F-actin but not of microtubules. (**a**) After the HG treatment that lasted three hours, cells were fixed and stained. Nuclei, stained with 4′,6-diamidino-2-phenylindole (DAPI, blue), of HT22 reference cells (kept on the centrifuge lid at 1× *g*, CTRL), and of cells centrifuged at 50× *g*. No morphological differences are evident. Scale bar: 10 µm. (**b**) Nuclear Area, Circularity, and Roundness are not different between centrifuged and reference cells. Roundness and Circularity were measured as described in the Methods section. Data represent mean ± SEM. Statistical test: Student’s *t*-test for unpaired groups (ns = not significant). (**c**) Left: representative images of F-Actin staining of reference (CTRL) and treated (50× *g*) cells (Phalloidin). Scale bar: 10 µm. Center: fluorescence was analyzed with Plot Profile (ImageJ suite); red curve: samples at 50× *g*; black curve: reference cells at 1× *g*. Right: data show an increase of relative fluorescence in centrifuged samples associated with a structural reorganization of fibers. Data represent mean ± SEM. Statistical test: Student’s *t*-test for unpaired groups. * = *p* < 0.05. (**d**) Left: representative images of microtubules (IF with anti-beta-tubulin antibodies) of HT22 reference cells (CTRL) and cells centrifuged at 50× *g*. Scale bar: 10 µm. Center: fluorescence intensity was analyzed with Plot Profile (ImageJ suite). Red lines: 50× *g*; black lines: reference cells at 1× *g*. Right: data do not show any increase in fluorescence intensity. Data represent mean ± SEM. Statistical test: Student’s *t*-test for unpaired groups. ns = not significant. *n* = 3 independent biological replicas (i.e., number of experiments), each one having at least three technical replicas. For each replica, at least 20 nuclei were analyzed, and at least 20 cells were analyzed for actin and tubulin staining.

Changes in gravitational loading create mechanical stress that may affect nuclear and cell morphology. To assess that, we measured the Area, Circularity, and Roundness of nuclei of HT22 cells treated as in Figure 1a. The results revealed no significant differences in any of the parameters (Figure 2a,b), indicating that three hours at 50× *g* did not affect such morphological parameters in centrifuged cells. Also, 50× *g* increased the fluorescence intensity of F-actin and changed the shape of the fluorescence curves (Figure 2c), suggesting a macroscopic rearrangement of microfilaments. The areas under the red and black curves are different, indicating a weaker fluorescence intensity of the control cells (black curve) compared to the centrifuged cells (red curve). The quantitative analysis in the histogram on the right side confirms the interpretation of the plot: the relative fluorescence measurement shows a statistically significant increase (* = *p* < 0.05, Student *t*-test for unpaired samples) in centrifuged cells over control cells. The different indentation of the two curves indicates a different occurrence of stress fiber thickness [28].

No changes were found for microtubules (Figure 2d). We concluded that HG does not affect nuclear morphology and microtubule patterns but affects the F-actin dynamics in HT22 cells.

**Figure 3:**
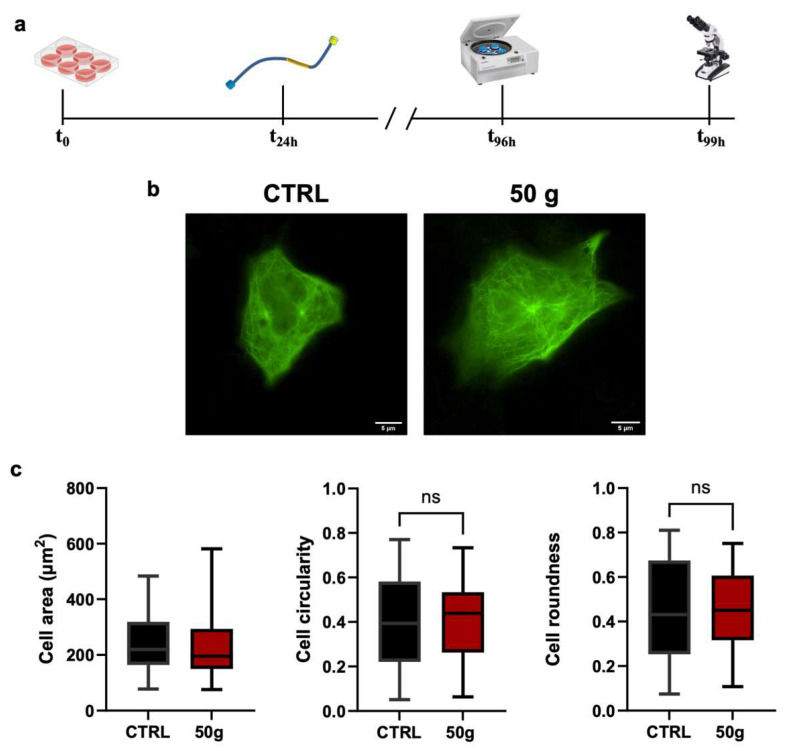
HT22 cells transfected with CST sensor allow visualization of microtubules and endure HG. Tau is an intrinsically disordered protein that, like other natively unfolded proteins, tends to be highly flexible [13]. From being distended when free in the cytosol, Tau shifts to a hairpin structure when bound to microtubules [13]. This change prompted researchers to develop a conformational-sensitive Tau (CST) sensor for measuring the ratio of microtubule-bound Tau versus free Tau in the cytoplasm or aggregated into NFTs. The tool, originally developed by co-authors of this work, takes advantage of a full-length Tau protein carrying two different, terminal fluorophores that work as a FRET couple [29]. (**a**) Experimental design: cells were seeded, allowed to attach and spread, and transfected with the CST sensor (which emits green fluorescence when excited at 500 nm). Microtubules are visualized in detail because of their binding with CST. 96 h from seeding, cells were centrifuged for three hours and immediately afterward fixed and analyzed under the microscope. (**b**) Results showed that the cells, both reference (that remained on the centrifuge lid, CTRL) and centrifuged at 50× *g*, maintained their characteristic elongated and branched shape. All recombinant Tau protein was associated with microtubules; no intracellular deposits were observed. The areas of highest fluorescence correspond to the microtubule organizing centers (MTOCs), which are known to contain a dense concentration of Tau protein. Scale bar: 5 µm. (**c**) Area, Circularity, and Roundness of live, CST-transfected HT22 cells did not change between CTRL and centrifuged cells. Data represent mean ± SEM. Statistical test: groups were compared with Student’s *t*-test for unpaired samples, *n* > 45; ns = not significant. *n* = 3 independent biological replicas (i.e., number of experiments), each one with at least three technical replicas. For each replica, at least 20 cells were analyzed.

Next, we transfected HT22 cells with the Conformational Sensitive Tau (CST) sensor to study adaptive changes of Tau aggregation to HG. CST allows for the real-time visualization of Tau in living cells due to the presence of a Fluorescence Resonance Energy Transfer (FRET) couple at the N-terminus and C-terminus of the Tau protein [29] (Figure 3a). We measured the Area and morphology of CST-positive cells before and after three hours of centrifugation (Figure 3b). The results showed no significant changes in Circularity, Roundness, or cell Area between the CST-positive cells exposed to 50× *g* and controls (Figure 3c), confirming that the expression of the CST sensor did not alter the morphological parameters in HG and control cells.

**Figure 4:**
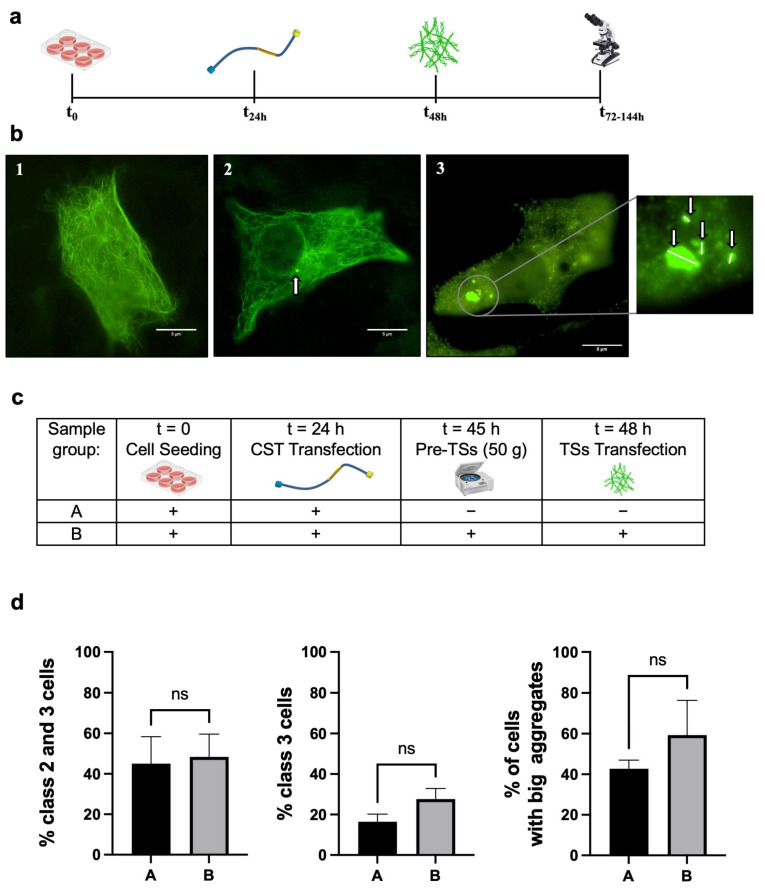
Mouse hippocampal cells HT22 transfected with Tau seeds undergo Tau aggregation. (**a**) Experimental design to evaluate the feasibility of double transfection in HT22 cells, to visualize Tau conformational changes and induce neurofibrillary tangles (NFTs) deposition, as a model of neurodegeneration: cells were seeded, allowed to attach and spread; 24 h later, cells were transfected with CST sensor. 48 h after seeding, cells were also transfected with TSs. Different batches of cells were fixed and analyzed under the microscope up to 5 days after TSs transfection, for a time course evaluation of the phenotype. (**b**) Analysis of the phenotypes of Tau aggregates (indicated by arrows). CST—positive, TS—transfected cells were divided into three classes based on the phenotype: Class 1, cells expressing CST, with clearly detectable microtubules and no visible aggregates (**left**); Class 2: cells displaying a small number of aggregates, with CST still visible in the microtubule lattice (**center**); Class 3: cells exhibiting a high number of aggregates, with microtubules no longer detectable in the cytoskeleton (**right**). The threshold used to divide aggregates into major and minor was 0.16 μm^2^, which corresponds to the 75th percentile of the reference group (Sample A, see below). Scale bar: 5 µm. Inset: magnification of Tau aggregates. (**c**) Experimental design to evaluate a potential protective effect of HG (50× *g*) from the onset of degeneration (i.e., Tau precipitation). Cells were prepared as above, except for a three-hour centrifugation at 50× *g* before TS transfection (Pre—TSs, which was performed right at the end of the centrifugation). Cells were observed 24 h later. (**d**) Results showed that the relative percentage of cells with major Tau aggregates did not change as a result of the Pre−TSs 50× *g*, suggesting in this scenario, HG does not prevent the onset of Tau precipitation. Data represent mean ± SEM. Statistical test: groups were compared with Student’s *t*-test for unpaired samples, n > 60; ns = not significant. *n* = 3 independent biological replicas (i.e., number of experiments), each one having its own technical replicas in the number of at least 3, for each experimental condition. For each technical replicate, at least 30 cells were analyzed.

Next, we induced a neurodegenerative phenotype characterized by Tau aggregation by transfecting CST-positive HT22 cells with synthetic Tau neurofibrillary tangle seeds (TSs, experimental design in Figure 4a). The treatment produced phenotypes that, with a phenotypic screening approach, we divided into Classes 1 to 3 (Figure 4b). Class 3 was considered the most degenerated phenotype.

To determine whether 50× *g* could prevent the precipitation of aggregates induced by TS transfection, we centrifuged HT22 cells before the transfection of TSs (referred to as Pre-TSs HG). We then counted the percentage of CST-positive cells exhibiting Tau aggregates (experimental design in Figure 4c; samples are labeled with letters for clarity) and found that 50× *g* did not affect the percentage of cells with aggregates of any size (Classes 2 and 3 combined, Figure 4d left). Next, we asked whether Pre-TSs HG would influence the proportion of Class 3 cells (highly degenerated phenotype) relative to cells with Tau aggregates of any size, and found this is not the case (Figure 4d center). Additionally, since the size of pathological inclusions of Tau can vary significantly (Figure 4b), we assessed whether Pre-TSs HG could modulate the Area of Tau aggregates in HT22 CST-positive, TS-transfected cells. We found that the treatment (Figure 4c) did not change the percentage of cells with aggregate sizes exceeding the 75th percentile of the reference group (Group A) (Figure 4d, right). Overall, we concluded that Pre-TS HG does not change the percentage of cells with Tau aggregates, their numerosity, or Area.

**Figure 5:**
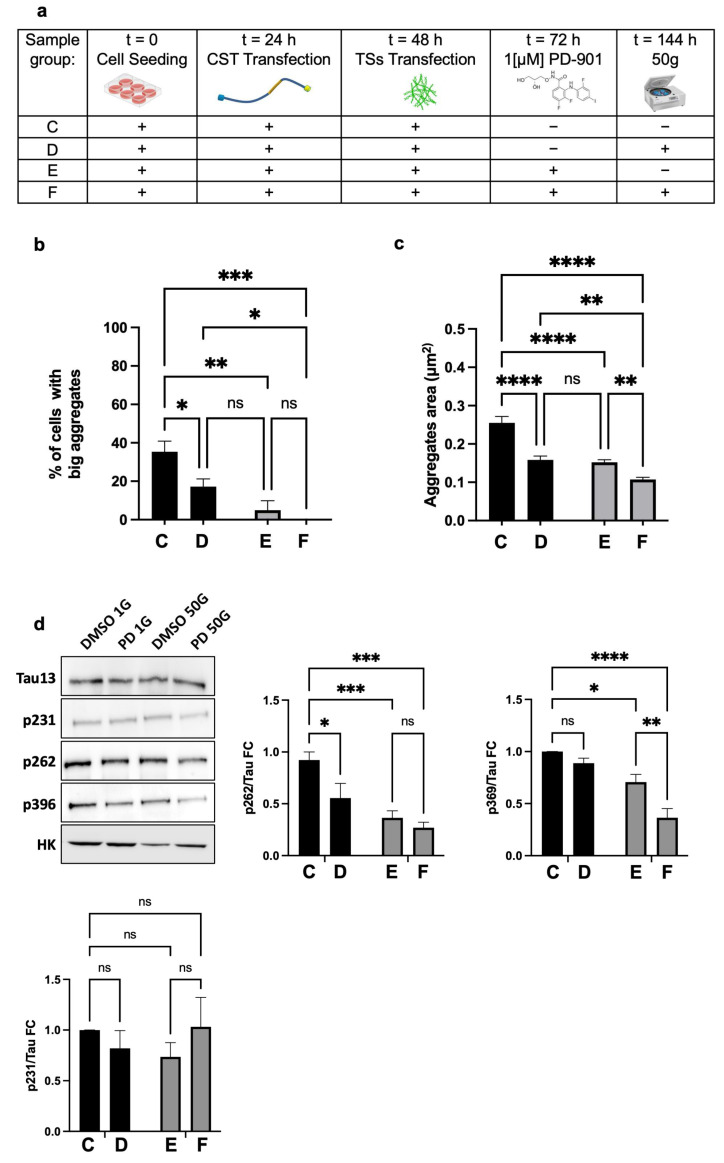
HG alone, and synergistically when combined with ERK inhibition, significantly reduce pathological Tau phosphorylation at specific, critical residues. (**a**) Experiment design: cells were seeded, allowed to attach and spread, transfected with CST, induced to Tau deposition, and treated with ERK inhibitor PD-901 (or vehicle). The treatment lasted for 72 h following Tau seed (TS) administration, which induced Tau aggregation. Centrifugation at 50× *g* for 3 h was applied afterwards. (**b**) Centrifuged cells (Group D) showed fewer cells with large Tau aggregates, suggesting that HG effectively countered the most aggressive phenotype of Tau aggregation. When HG was combined with PD-901 (Group F), the reduction was even stronger, suggesting that the two treatments have a synergistic effect; although there was no statistical difference, we noticed that cells treated with ERK-inhibitor and HG combined showed no Tau aggregates above the threshold in any of the biological replicas. (**c**) The synergistic effect of the combined HG and drug administration was also mirrored by the statistically significant reduction of aggregate Area. (**d**) The morphological observations described above were confirmed by the biochemical analysis: the reduction of p626 was evident after centrifugation, and even more marked after the combination of HG and PD-901. Reduction of p231 was more pronounced after the combined treatments, suggesting that HG alone is not as effective as PD-901 in reducing phosphorylation of this residue. Interestingly, p231 was not modified by either treatment, suggesting a specificity of action of both treatments. Data represent mean ± SEM. Statistical test: groups were compared using one-way ANOVA with Tukey’s multiple comparison post-hoc test. With “*n*” indicating the number of independent biological replicas, the samples’ size was as follows: *n* = 6 for p262; *n* = 7 for p231; *n* = 5 for p396. ns = not significant, * = *p* < 0.05, ** = *p* < 0.01, *** = *p* < 0.001, **** *p* < 0.0001.

Thereafter, to determine whether the combination of HG and PD-901 could rescue the degenerated phenotype, we combined centrifugation at 50× *g* with the administration of 1µM PD-901, on NFT-positive cells (experimental design in Figure 5a).

We assessed the percentage of cells with larger aggregates, setting the threshold at the 75th percentile of the reference group (Group C, Figure 5a). We found that Group D (50× *g*) or Group E (PD-901) had significantly fewer cells exceeding the threshold and, interestingly, without significant difference between the two groups (D and E). However, cells that underwent the combined treatments (Group F) showed no aggregates above the threshold (Figure 5b). These data strongly suggested that 50× *g* or PD-901 are almost equally effective in reducing the formation of larger Tau aggregates.

Regarding the Area of CST aggregates, the mean Area in Groups C and D was significantly lower compared to controls. Notably, the combination of HG and PD-901 (Group F) led to a further significant reduction in aggregate Area against controls (Figure 5c). We concluded that 50× *g* alone significantly reduced the number of cells with larger Tau aggregates and decreased the overall Tau aggregate Area. Furthermore, the inhibition of ERK activity and HG worked synergistically to reduce the formation of Tau intracellular inclusions.

The rearrangement of F-actin caused by HG likely influences the availability of ERK. Since Tau phosphorylation strongly associates with its pathological aggregation, we used Western blot to analyze in HT22 cells the phosphorylation of residues that are relevant to Tau destabilization/aggregation. As expected [17], we found that PD-901 alone significantly reduced the phosphorylation of S262 and S396 (Figure 5d), both of which are key residues located in the microtubule-binding domain and C-terminal regions that facilitate the formation of pathological inclusions. Interestingly, 50× *g* alone caused a notable decrease in pS262 but did not significantly affect pS396, indicating a lower efficacy in preventing the phosphorylation of this critical residue. However, the combination of PD-901 and HG led to a substantial reduction in both pS262 and pS396 (about 65–75% compared to control cells), which was significantly greater than what was observed with either treatment alone. This suggests that the two treatments work synergistically to prevent Tau phosphorylation, thereby reducing aggregation. Noticeably, the experimental conditions above did not affect the phosphorylation of Tau at pT231, suggesting they target specific pathological residues. We concluded that HG and ERK inhibition combined significantly reduce pathological Tau phosphorylation at specific, critical residues.

**Figure 6:**
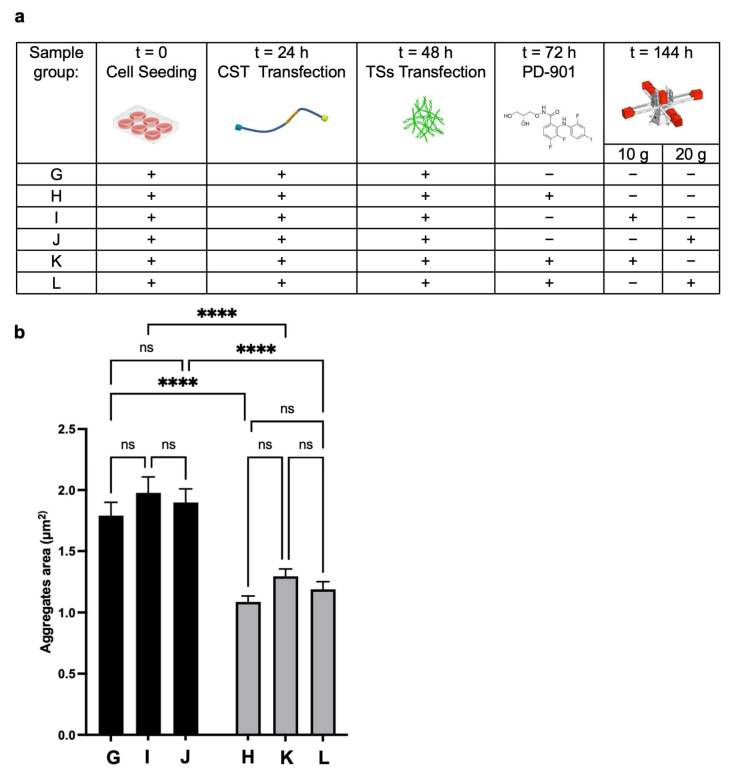
HG experiment at the LDC facility (ESA-ESTEC, Noordwijk, The Netherlands). (**a**) HT22 cells were seeded, transfected with the CST sensor, afterwards with TS, then treated with the ERK inhibitor PD-901, or vehicle, and centrifuged at 10× *g* or 20× *g*. (**b**) As expected, cells exposed to PD-901 (gray) showed aggregates with smaller Area compared to vehicle (black). However, the centrifugation per se did not cause any change. Data represent mean ± SEM. Statistical test: one-way ANOVA with Tukey’s multiple comparison post hoc test. ns = not significant. **** = *p* < 0.0001. n = 3 independent biological replicas (i.e., number of experiments), each one having at least 3 technical replicates. For each replica, at least 35 cells were analyzed.

To study the effects of lower g-values, we performed an experimental campaign at the LDC, testing 10× *g* and 20× *g* that cannot be reliably produced with standard lab centrifuges, along with other characteristics of the run, such as minimal shear stress [27]. We evaluated the impact of lower HG alone and combined with PD-901 (experimental design in Figure 6a) on the Area of CST aggregates.

Tau inclusions were present in CST-positive reference cells (Group G). As expected, the aggregate Area was significantly reduced in cells treated with the drug (Group H) compared to the control (Figure 6b); however, it was not smaller in cells from Group I and Group J (exposed to 10× *g* and 20× *g*). Coherently, cells exposed to the combined treatments (PD-901 and 10× *g* or 20× *g*) did not exhibit significant differences in aggregate area compared to cells treated with the drug alone, suggesting that 10× *g* and 20× *g* do not have an independent effect. We propose that there is a potential threshold effect of gravitational force in modulating CST aggregate formation and Area in the in vitro model, even when combined with the ERK inhibitor.

## 4. Discussion

**Figure 7:**
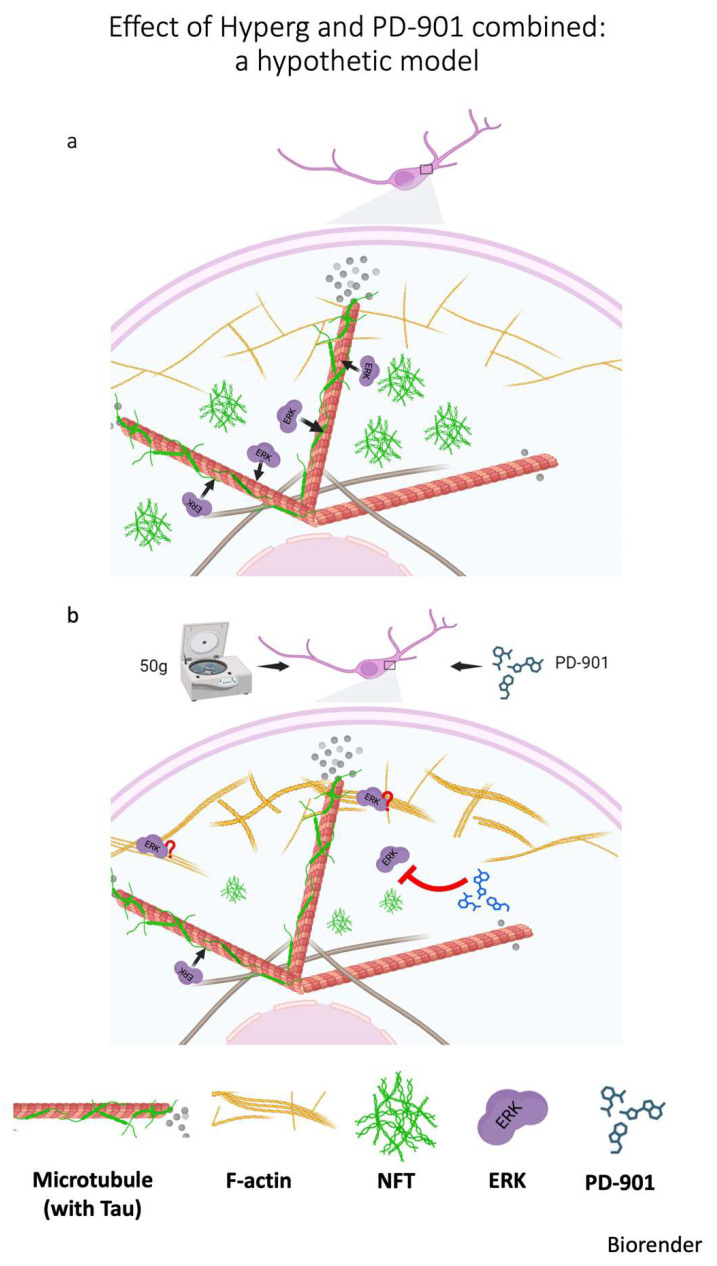
A hypothetical model of the effect of HG on Tau deposition. (**a**) In a neuronal degenerative model of NFT deposition, the ERK kinase has access to TAU associated with microtubules, causing further NFT deposition. (**b**) Centrifugation promotes thickening of actin fibers, sequestering ERK. The drugs synergize with this effect [32]. The red question mark suggests the strengthened association of ERK with F-actin upon HG treatment. The red T–bar indicates inhibition of PD–901 on ERK activity.

Here, we present critical insights into the impact of HG on neuronal cell behavior in the context of tauopathy. We show that hippocampal neuronal cells can withstand HG without compromising cell viability or altering cell/nuclear morphology, which lays the groundwork for further investigations into the effects of gravitational changes on neuronal pathophysiology. Our results with the CST sensor further elucidate the cellular response to HG. The combined analyses of Tau aggregation post-TS transfection indicated that pre-exposure to HG does not mitigate Tau aggregation, which may counter some expectations regarding gravitational force acting as a protective mechanistic factor against neurodegeneration [30]. Despite the lack of an observable preventive effect of HG on Tau aggregates, our subsequent experiments demonstrated that the combination of 50× *g* and the ERK inhibitor PD-901 significantly reduces the formation of larger Tau aggregates while concurrently decreasing the pathological phosphorylation of Tau at key residues. This finding presents a compelling case for the synergistic action of HG and pharmacological intervention to mitigate Tau-related pathology. A hypothetical model is proposed in Figure 7. It is known that ERK localizes in distinct regions within the cell, also associating with F-actin that can serve as a scaffold protein, thereby affecting ERK availability and activity [31]. Thicker actin bundles, as a consequence of HG, likely hinder the accessibility of ERK to its substrates, among which Tau protein. The hypothesis relates to the spatial organization and density of actin: Thicker actin bundles may sequester ERK away from its target substrates, leading to reduced phosphorylation of Tau. This sequestration could be due to the physical barrier that the dense actin structures create, limiting ERK movement within the cytoplasm and its interaction with Tau. Or, thick bundles might contribute to altered mechanical properties and cell signaling dynamics, which in turn could influence ERK distribution. Consequently, specific cellular stressors that promote thicker actin filaments may create conditions that downregulate ERK’s ability to phosphorylate Tau. Noticeably, activation of ERK on stress fibers increases with the magnitude of the tensile forces acting on the fibers [32]; furthermore, HG causes a rearrangement of F-actin, producing thicker stress fibers [18]. So, HG could potentially impact ERK/Tau interaction, which is critical in neurodegeneration [33]. Therefore, understanding the interplay between ERK and actin architecture in HG may provide insights into cellular signaling and conditions affecting Tau hyperphosphorylation in neurodegeneration.

The observation that pre-TSs do not protect against degeneration induced by TSs suggests that, in this experimental setting, centrifugation before Tau aggregation might not effectively mimic the cellular or molecular conditions present during and after the initiation of Tau-related pathology.

The interplay between mechanical forces, cell stress-response, and Tau aggregation mechanisms is complex and highlights the significance of timing in addition to the specific cell context [34,35]. Further investigations will elucidate why the timing of HG exposure yields different effects on Tau pathology.

Furthermore, the observation that HG affects F-actin without significantly impacting microtubule organization or nuclear morphology is intriguing. It suggests a selective mechanical response of the cytoskeleton, potentially modulating cellular functions crucial for maintaining neuronal health, thereby paving the way for studies into the mechanotransductive pathways involved.

Moreover, the characterization of the different classes of Tau aggregates provides valuable information on the cell state in terms of disease progression. The classification system based on aggregate “presence” and “morphology” enables a more nuanced discussion regarding stages of tauopathy and responses to therapeutic modalities. The negligible difference in aggregation between the groups treated with PD-901 and those subjected to HG alone underscores the need for further exploration into the molecular mechanisms driving Tau phosphorylation and aggregation.

Interestingly, the experimental results from the lower gravitational conditions (10× *g* and 20× *g*) suggest a threshold effect of gravitational forces on Tau pathophysiology, where higher gravitational forces may be necessary to instigate significant changes in Tau behavior in vitro.

On this basis, it is essential to consider the broader implications for therapeutic strategies targeting tauopathies. Our results suggest that innovative approaches may integrate mechanical forces, such as HG, with pharmacological agents to enhance therapeutic efficacy. Additionally, studies exploring more extensive ranges of HG values and their impact on neuronal plasticity and integrity could potentially lead to new therapeutic modalities compatible with treatments for human subjects. In fact, it was proposed that HG exerts a hormetic effect, meaning it is a stressor that, in low amounts or for a short period of time, might exert beneficial/protective effects [36]. However, the underpinning molecular mechanisms are still completely unknown.

## 5. Conclusions and Study Limitations

This study, for the first time, sheds light on the complex interplay between mechanical forces and intracellular signaling pathways that underpin Tau phosphorylation and aggregation. The demonstrated protective effect of combining HG with ERK inhibition offers a promising avenue for developing novel interventions for tauopathies. Further in vivo studies and research using humanized models are warranted to elucidate the underlying molecular mechanisms. In fact, our hypothesis (in Figure 7)—that combined treatments with HG and drug may cause denser or thicker F-actin structures to physically interfere with the movement or distribution of ERK—has not been directly demonstrated in this study, and this represents a limitation. However, this hypothesis is supported by substantial evidence from the literature indicating that, in various systems, ERK and F-actin physically interact [37,38,39]. F-actin is required for the formation of signaling complexes where pERK co-localizes with F-actin and other signaling components; disrupting actin polymerization prevents pERK stabilization [40]. Collectively, evidences support a model whereby F-actin plays dual roles depending on its stabilization status: it can serve as a scaffold and localize ERK, facilitating or restricting its phosphorylation, depending on the context; or, when disrupted, it can release ERK, altering both the level and localization of its activation [40]. Supporting this, in a smooth muscle cell line, the association of ERK1/2 with the actin cytoskeleton influences ERK1/2 signaling outcome, indicating that this interaction plays a regulatory role [41].

Furthermore, treatments with actin-disrupting agents such as cytochalasin D [41] or latrunculin B [39] significantly attenuated ERK phosphorylation, suggesting that an intact actin cytoskeleton is essential for optimal ERK activation. Future experiments will explore whether combining HG and PD-901 treatment with actin polymerization inhibitors can reverse HG-induced effects on Tau phosphorylation. Additionally, the co-immunoprecipitation of actin and ERK proteins following HG treatment could help confirm a direct physical interaction in this context.

Addressing this gap will be a focus of future research efforts.

## Data Availability

The original contributions presented in this study are included in the article/Supplementary Materials. Further inquiries can be directed to the corresponding author.

## Supplementary Materials

The following supporting information can be downloaded at: https://www.mdpi.com/article/10.3390/cells14141058/s1.

## List of Abbreviations

CST	Conformational Sensitive Tau sensor
DAPI	4′,6-diamidino-2-phenylindole
DMSO	Dimethyl sulfoxide
EEG	electroencephalogram
ERK	Extracellular signal-Regulated Kinase
ESA	European Space Agency
ESTEC	European Space Research and Technology Center
FRET	Fluorescence Resonance Energy Transfer
HG	hypergravity
IF	Immunofluorescence assay
LDC	Large Diameter Centrifuge
MG	microgravity
NFT	neurofibrillary tangles
PBS	phosphate buffer solution
PFA	paraformaldehyde
RT	room temperature
SEM	standard error of the mean
TS	Tau seed

## References

[1] Clément G., Wood S.J., Reschke M.F. (1992). Effects of microgravity on the interaction of vestibular and optokinetic nystagmus in the vertical plane. Aviat. Space Environ. Med..

[2] Roberts D.R., Albrecht M.H., Collins H.R., Asemani D., Chatterjee A.R., Spampinato M.V., Zhu X., Chimowitz M.I., Antonucci M.U. (2017). Effects of Spaceflight on Astronaut Brain Structure as Indicated on MRI. N. Engl. J. Med..

[3] Garrett-Bakelman F.E., Darshi M., Green S.J., Gur R.C., Lin L., Macias B.R., McKenna M.J., Meydan C., Mishra T., Nasrini J. (2019). The NASA Twins Study: A multidimensional analysis of a year-long human spaceflight. Science.

[4] McGregor H.R., Hupfeld K.E., Pasternak O., Beltran N.E., De Dios Y.E., Bloomberg J.J., Wood S.J., Mulavara A.P., Riascos R.F., Reuter-Lorenz P.A. (2023). Impacts of spaceflight experience on human brain structure. Sci. Rep..

[5] Acharya M.M., Baulch J.E., Klein P.M., Baddour A.A.D., Apodaca L.A., Kramár E.A., Alikhani L., Garcia C., Angulo M.C., Batra R.S. (2019). New Concerns for Neurocognitive Function during Deep Space Exposures to Chronic, Low Dose-Rate, Neutron Radiation. eNeuro.

[6] Mao X.W., Nishiyama N.C., Byrum S.D., Stanbouly S., Jones T., Holley J., Sridharan V., Boerma M., Tackett A.J., Willey J.S. (2020). Spaceflight induces oxidative damage to blood-brain barrier integrity in a mouse model. FASEB J..

[7] Wang H., Liu J., Lu Z., Dai Y., Xie J., Xu S., Song Y., Xiao G., Gao F., Qu L. (2020). Implanted multichannel microelectrode array for simultaneous electrophysiological signal detection of hippocampal CA1 and DG neurons of simulated microgravity rats. Biochem. Biophys. Res. Commun..

[8] Chattopadhyay M., Durazo A., Se H.S., Strong C.D., Gralla E.B., Whitelegge J.P., Valentine J.S. (2008). Initiation and elongation in fibrillation of ALS-linked superoxide dismutase. Proc. Natl. Acad. Sci. USA.

[9] Krzek M., Stroobants S., Gelin P., Malsche W.D., Maes D. (2022). Influence of Centrifugation and Shaking on the Self-Assembly of Lysozyme Fibrils. Biomolecules.

[10] Demontis G.C., Germani M.M., Caiani E.G., Barravecchia I., Passino C., Angeloni D. (2017). Human Pathophysiological Adaptations to the Space Environment. Front. Physiol..

[11] Cialdai F., Brown A.M., Baumann C.W., Angeloni D., Baatout S., Benchoua A., Bereiter-Hahn J., Bottai D., Buchheim J.-I., Calvaruso M. (2023). How do gravity alterations affect animal and human systems at a cellular/tissue level?. NPJ Microgravity.

[12] Davis T., Tabury K., Zhu S., Angeloni D., Baatout S., Benchoua A., Bereiter-Hahn J., Bottai D., Buchheim J.-I., Calvaruso M. (2024). How are cell and tissue structure and function influenced by gravity and what are the gravity perception mechanisms?. NPJ Microgravity.

[13] Kovacs G.G. (2017). Tauopathies. Handb. Clin. Neurol..

[14] Frost B. (2023). Alzheimer’s disease and related tauopathies: Disorders of disrupted neuronal identity. Trends Neurosci..

[15] Creekmore B.C., Watanabe R., Lee E.B. (2024). Neurodegenerative Disease Tauopathies. Annu. Rev. Pathol. Mech. Dis..

[16] Zhang X., Wang J., Zhang Z., Ye K. (2024). Tau in neurodegenerative diseases: Molecular mechanisms, biomarkers, and therapeutic strategies. Transl. Neurodegener..

[17] Siano G., Caiazza M.C., Ollà I., Varisco M., Madaro G., Quercioli V., Calvello M., Cattaneo A., Primio C.D. (2019). Identification of an ERK Inhibitor as a Therapeutic Drug Against Tau Aggregation in a New Cell-Based Assay. Front. Cell. Neurosci..

[18] Barravecchia I., Cesari C.D., Forcato M., Scebba F., Pyankova O.V., Bridger J.M., Foster H.A., Signore G., Borghini A., Andreassi M. (2022). Microgravity and space radiation inhibit autophagy in human capillary endothelial cells, through either opposite or synergistic effects on specific molecular pathways. Cell. Mol. Life Sci..

[19] Cesari C.D., Barravecchia I., Pyankova O.V., Vezza M., Germani M.M., Scebba F., van Loon J.J.W.A., Angeloni D. (2020). Hypergravity Activates a Pro-Angiogenic Homeostatic Response by Human Capillary Endothelial Cells. Int. J. Mol. Sci..

[20] Isasi E., Isasi M.E., van Loon J.J.W.A. (2022). The application of artificial gravity in medicine and space. Front. Physiol..

[21] Wade N.J., Norrsell U., Presly A. (2005). Cox’s chair: “A moral and a medical mean in the treatment of maniacs”. Hist. Psychiatry.

[22] Breathnach C.S. (2010). Hallaran’s circulating swing. Hist. Psychiatry.

[23] Kourtidou-Papadeli C., Frantzidis C.A., Bakirtzis C., Petridou A., Gilou S., Karkala A., Machairas I., Kantouris N., Nday C.M., Dermitzakis E.V. (2022). Therapeutic Benefits of Short-Arm Human Centrifugation in Multiple Sclerosis–A New Approach. Front. Neurol..

[24] Takamatsu Y., Koike W., Takenouchi T., Sugama S., Wei J., Waragai M., Sekiyama K., Hashimoto M. (2016). Protection against neurodegenerative disease on Earth and in space. NPJ Microgravity.

[25] Cairns N.J., Lee V.M.-Y., Trojanowski J.Q. (2004). The cytoskeleton in neurodegenerative diseases. J. Pathol..

[26] Eira J., Silva C.S., Sousa M.M., Liz M.A. (2016). The cytoskeleton as a novel therapeutic target for old neurodegenerative disorders. Prog. Neurobiol..

[27] Loon J.J.W.A.V., Folgering E.H.T.E., Bouten C.V.C., Veldhuijzen J.P., Smit T.H. (2003). Inertial shear forces and the use of centrifuges in gravity research. What is the proper control?. J. Biomech. Eng..

[28] Marino A., Filippeschi C., Genchi G.G., Mattoli V., Mazzolai B., Ciofani G. (2014). The Osteoprint: A bioinspired two-photon polymerized 3-D structure for the enhancement of bone-like cell differentiation. Acta Biomater..

[29] Di Primio C., Quercioli V., Siano G., Rovere M., Kovacech B., Novak M., Cattaneo A. (2017). The Distance between N and C Termini of Tau and of FTDP-17 Mutants Is Modulated by Microtubule Interactions in Living Cells. Front. Mol. Neurosci..

[30] Hashimoto M., Ho G., Shimizu Y., Sugama S., Takenouchi T., Waragai M., Wei J., Takamatsu Y. (2018). Potential Application of Centrifuges to Protect the CNS in Space and on Earth. Curr. Alzheimer Res..

[31] Mendoza M.C., Vilela M., Juarez J.E., Blenis J., Danuser G. (2015). ERK reinforces actin polymerization to power persistent edge protrusion during motility. Sci. Signal..

[32] Hirata H., Gupta M., Vedula S.R.K., Lim C.T., Ladoux B., Sokabe M. (2015). Actomyosin bundles serve as a tension sensor and a platform for ERK activation. EMBO Rep..

[33] Khezri M.R., Yousefi K., Esmaeili A., Ghasemnejad-Berenji M. (2023). The Role of ERK1/2 Pathway in the Pathophysiology of Alzheimer’s Disease: An Overview and Update on New Developments. Cell. Mol. Neurobiol..

[34] Lu H.P. (2023). A Missing Origin of the Tau Protein Aggregation Pathway Triggered by Thermal and Biological Forces. J. Integr. Neurosci..

[35] Hernández F., Ferrer I., Pérez M., Zabala J.C., del Rio J.A., Avila J. (2022). Tau Aggregation. Neuroscience.

[36] Minois N. (2006). The hormetic effects of hypergravity on longevity and aging. Dose Response Publ. Int. Hormesis Soc..

[37] Dugina V., Shagieva G., Khromova N., Kopnin P. (2018). Divergent impact of actin isoforms on cell cycle regulation. Cell Cycle Georget. Tex..

[38] Würtemberger J., Tchessalova D., Regina C., Bauer C., Schneider M., Wagers A.J., Hettmer S. (2020). Growth inhibition associated with disruption of the actin cytoskeleton by Latrunculin A in rhabdomyosarcoma cells. PLoS ONE.

[39] Tomas A., Yermen B., Min L., Pessin J.E., Halban P.A. (2006). Regulation of pancreatic beta-cell insulin secretion by actin cytoskeleton remodelling: Role of gelsolin and cooperation with the MAPK signalling pathway. J. Cell Sci..

[40] Singhai A., Wakefield D.L., Bryant K.L., Hammes S.R., Holowka D., Baird B. (2014). Spatially Defined EGF Receptor Activation Reveals an F-Actin-Dependent Phospho-Erk Signaling Complex. Biophys. J..

[41] Vetterkind S., Saphirstein R.J., Morgan K.G. (2012). Stimulus-Specific Activation and Actin Dependency of Distinct, Spatially Separated ERK1/2 Fractions in A7r5 Smooth Muscle Cells. PLoS ONE.

