# Supplementary material for: Hypergravity and ERK Inhibition Combined Synergistically Reduce Pathological Tau Phosphorylation in a Neurodegenerative Cell Model
Source: Cells. 2025 Jul 10;14(14):1058. doi: 10.3390/cells14141058 (PMC12293720; doi:10.3390/cells14141058)
Supplement: Supplementary file 1 [file cells-14-01058-s001.zip › cells-3576210-supplementary.pdf]

Supplementary Materials

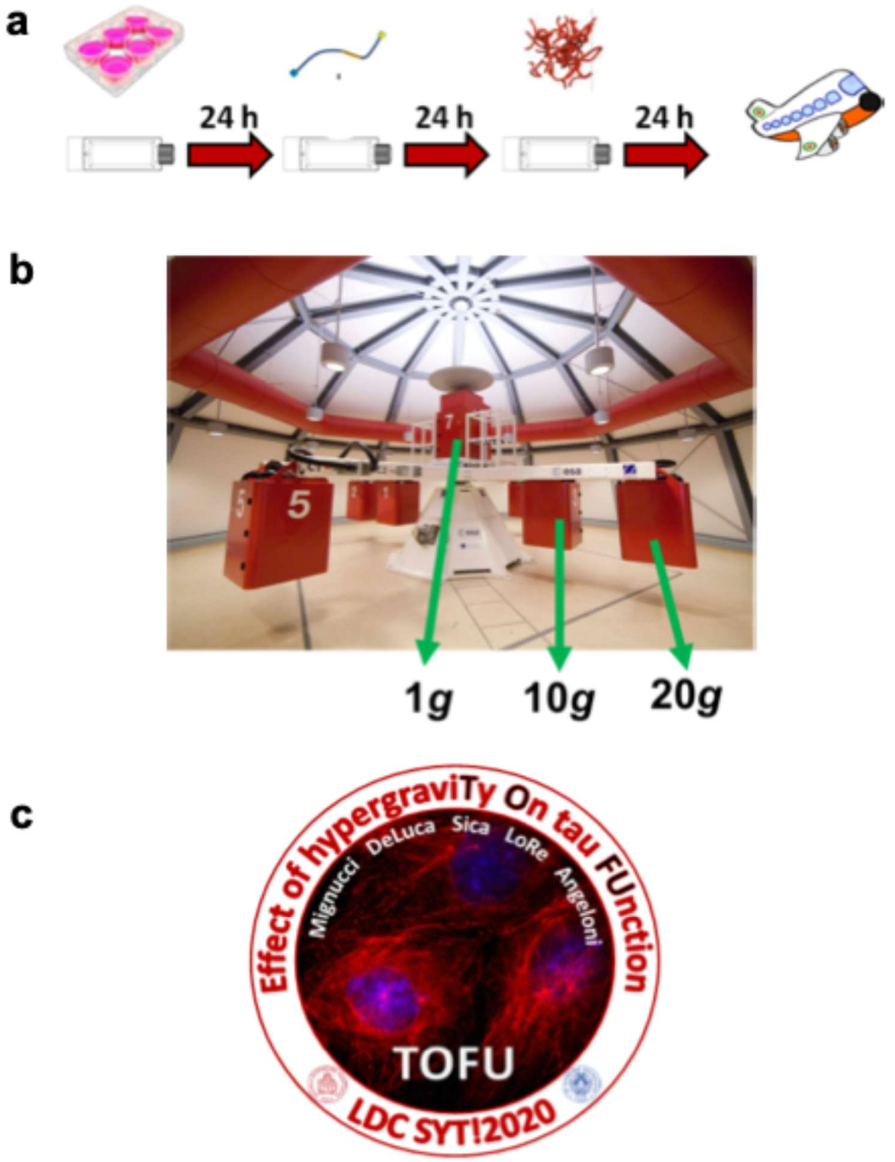

Scheme S1. The Large Diameter Centrifuge. We had the Large Diameter Centrifuge (LDC) facility (European Space Research and Technology Center (ESTEC) of ESA (Noordwijk, ND), accessible to us for a two-and-a-half-day session, through a competitive selection, after responding to a Spin your thesis! call from ESA. a) Design of the protocol performed for the experiment at LDC. Cells were seeded, transfected with CST, and induced with TSs as in Figure 4A, and then flown to the LDC laboratory. b) The LDC is comprised of four arms, holding in total six swinging gondolas, with a diameter of 8m at full swing-out. The HG field inside the gondolas is generated by the centripetal forces due to

rotation; at regime, the acceleration vector is perpendicular to the sample surface, thereby eliminating shear stress. There is an additional gondola, which serves as a reference, fixed in the center, exposed to vibrations generated by the centrifuge but not to gravitational acceleration. 20g is the LDC upper limit. Photo credit: ESA. c) Mission patch of the 'Effect of HG on Tau function (TOFU)' study.

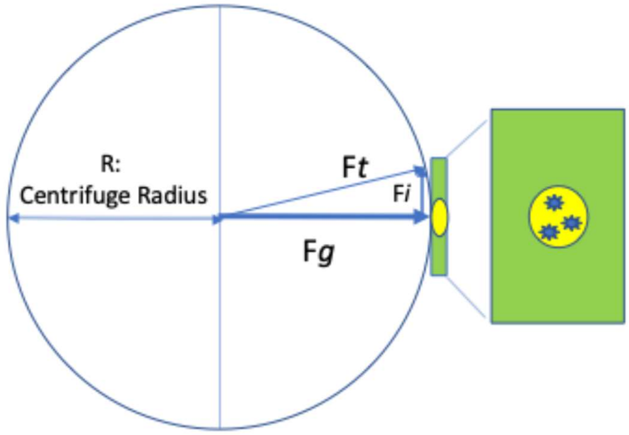

Scheme S2. Geometry of forces acting on the centrifuged cell culture. R: centrifuge radius is the distance from the center of rotation to the outer wall of the centrifuge. Fg: force of gravity. Fi: the inertial shear force, increases laterally from the center of centrifugation; Ft: the total resulting force. Scheme adapted from [17]. On the right: frontal-view representation of the cell container used: cells were seeded in a glass-bottom 3cm plate (yellow) fixed to the center of a rack in the swing-out rotor of the home standard centrifuge.
